# RBC, HB, HCT, CRP, and ESR at different postoperative periods after the application of intravenous unit dose transient acid in PLIF: A case control study

**DOI:** 10.3389/fsurg.2022.1032376

**Published:** 2023-01-06

**Authors:** Shenshen Hao, Xiangping Wang, Zenan Yue, Ruijun Zhang, Pengcheng Wang, Saike Meng, Shuai Liu, Hongke Li, Shengli Dong

**Affiliations:** ^1^ Department of Spine and Bone Oncology, General Hospital of Pingmei Shenma Medical Group, Pingdingshan, China; ^2^ Department of Anesthesia and Perioperative Medicine, General Hospital of Pingmei Shenma Medical Group, Pingdingshan, China; ^3^ Department of Theoretical Research Office, Party School of the CPC Pingdingshan Municipal Committee, Pingdingshan, China; ^4^ Medical Department, General Hospital of Pingmei Shenma Medical Group, Pingdingshan, China

**Keywords:** lumbar degenerative disease, posterior lumbar interbody fusion, tranexamic acid, anemia index, inflammatory response index

## Abstract

**Background:**

Tranexamic acid (TXA) has been used in posterior lumbar interbody fusion (PLIF) and reduces blood loss. However, it has not been reported whether it will continue to affect postoperative red blood cells (RBC), hemoglobin (HB), hematocrit (HCT), C-reactive protein (CRP) and erythrocyte sedimentation rate (ESR). The purpose of this study was to observed the above indicators at different time after PLIF with unit dose intravenous (iv) TXA.

**Methods:**

The data of 44 patients treated by single-segment PLIF from 2020.11 to 2022.3 were retrospectively analyzed. Observation group was given a unit dose of ivTXA (1 g/100 mL) 15 min before skin incision after general anesthesia. Patients without TXA were recorded as control group. Main observation indicators include RBC, HB, HCT, CRP and ESR on the 1st, 4th, 7th and last tested day after surgery. Secondary observation indicators include postoperative activated partial thrombin time (APTT), prothrombin time (PT), thrombin time (TT), and fibrinogen (FIB); and operation time, intraoperative blood loss, postoperative drainage volume, incision healing, postoperative deep vein thrombosis and postoperative hospital stay.

**Results:**

The operation was successfully completed without related complications. At term of main observation indicators, RBC, HB and HCT remained relatively stable, while CRP and ESR fluctuated to some extent after PLIF. The RBC, HB and HCT in the observation group were higher than those in the control group with statistically significant (*p* < 0.05). Except the CRP of 7th postoperative day of the observation group was significantly lower than that of the control group (*p* < 0.05), there was no difference in other CRP and ESR between the two groups (*p* > 0.05). At term of secondary observation indicators, the intraoperative blood loss and postoperative drainage volume of the observation group were lower than those of the control group with statistically significant (*p* < 0.05). There was no significant difference in postoperative APTT, PT, TT, FIB, and operation time and postoperative hospital stay between the two groups (*p* > 0.05).

**Conclusion:**

The application of unit dose of ivTXA in PLIF can safely and effectively reduce blood loss. Meanwhile, it can also maintain higher RBC, HB, HCT levels without disturbing CRP and ESR levels after surgery.

## Background

With the continuous increase of the elderly population and the improvement of patients' awareness of healthy life, the number of patients undergoing surgeries for lumbar degenerative diseases such as lumbar disc herniation, lumbar spinal stenosis, and lumbar spondylolisthesis has increased sharply in recent years (1, 2). Posterior lumbar interbody fusion (PLIF) is a common and effective operation for the treatment of lumbar degenerative diseases (3, 4). However, it faces the challenge of large perioperative blood loss (5). That can lead to anemia often requiring transfusion, which increases the risk of transfusion-related adverse events (6). Besides, PLIF is one of the major orthopaedic operations, which is also prone to infection after surgery, as a disaster.

Tranexamic acid (TXA), as a synthetic derivative of lysine, was discovered in 1962 and the chemical term was trans-4-aminomethylcyclohexanecarboxylic acid (7). It, as a synthetic antifibrinolytic drug, can reduce surgical bleeding by inhibiting fibrinolysis and stabilizing blood clots (8). Studies have reported that intravenous (iv)TXA can effectively reduce perioperative blood loss in PLIF (9–12). Red blood cell (RBC), hemoglobin (HB) and hematocrit (HCT) are commonly used clinical indicators to monitor the course of anemia. The current research basically focuses on HB, and only observed the value at a time point after surgery, such as the pioneer researcher Kushioka et al. (13). Therefore, this study plans to simultaneously and continuously observe the three indicators of RBC, HB, and HCT, in addition to intraoperative and posteperative blood loss.

The disaster that worries doctors the most after PLIF is infection. The commonly used indicators in inflammation monitoring after lumbar spine surgery are Serum procalcitonin, white blood cell count, C-reactive protein (CRP) and erythrocyte sedimentation rate (ESR), among which CRP has the highest specificity of 90.27%, and ESR has the highest sensitivity of 88.50% (14). Combined monitoring of CRP and ESR after lumbar spine surgery is important for monitoring infectious events (15). However, there are few reports on postoperative CRP and ESR monitoring with TXA in PLIF, and these are different for a single time point comparison (16, 17). Therefore, continuous monitoring of CRP and ESR after surgery may provide more information to know whether the ivTXA affects the judgment of inflammation after surgery.

There are various schemes for perioperative application of TXA, such as single preoperative intravenous administration, continuous intravenous infusion, topical administration, and intravenous combined topical administration (18, 19). Among them, the most used way is ivTXA (20). After about 15 min of ivTXA, it can reach and accumulate in the surgical field to exert hemostatic effect (21). Besides, this medication method is also the main recommendation for the application of TXA in the 2019 Chinese Expert Consensus, which has the advantage of not interfering with other intraoperative intravenous drugs (22). Single-level PLIF is one of the most common types of surgery. Therefore, in this study, the single-segment PLIF cases with ivTXA applied 15 min before surgery were selected as the research sample. This study mainly discusses two questions: (1) Describe the RBC, HB, HCT, CRP, and ESR after PLIF without TXA, and try to find out its characteristics. (2) Find out whether the above indexes are significantly affected after PLIF with TXA.

## Method

### Study design

As a case control study, the time frame for case collection was from 2020.11 to 2022.3, and the location was General Hospital of Pingmei Shenma Medical Group. The inclusion criteria included American Society of Anesthesiologists classification was grade II, anesthesia mode was general anesthesia, the surgical segment was single-segment PLIF, and there was no previous history of lumbar spine surgery. The exclusion criteria included those with deep venous thrombosis (DVT) before surgery, and those who received anticoagulation in coming 2 weeks before surgery, and those who had cerebrospinal fluid leakage or dural damage during surgery. In the end, 44 cases data was retrospectively collected, including 26 males and 18 females, with an average age of (55.57 ± 10.38) years old. Grouping was based on whether or not ivTXA was applied. Patients who started applying unit dose of ivTXA (1 g/100 mL) 15 min before skin incision after general anesthesia were included in the observation group. Patients who without applied TXA were included in the control group. The PLIF operation steps were the same and two drainage tubes were placed. When the patient returns to the ward after the operation, some conventional treatment measures are given. They included the application of cephalosporin antibiotics to prevent infection, glucocorticoids to reduce spinal cord stress response, glycerol drugs to reduce edema response, non-steroidal drugs to relieve pain, and low molecular weight heparin drugs to prevent DVT. The vital signs, surgical incision, sensation and movement of both lower extremities were observed. When the drainage volume is less than 50 mL/24 h, the drainage tubes will be removed. Meanwhile, preoperative general information of patients was collected as baseline data, including age, gender, body mass index (BMI), disease type, coexisting diabetes, coexisting high blood pressure, activated partial prothrombin time (APTT), prothrombin time (PT), thrombin time (TT), fibrinogen (FIB), RBC, HB, HCT, CRP and ESR.

### Outcome indicators

Main observation indicators include RBC, HB, HCT, CRP and ESR on the 1st, 4th, 7th and last tested day after surgery. Secondary observation indicators include APTT, PT, TT, FIB of the first postoperative day, and operation time, intraoperative blood loss, postoperative drainage volume, incision healing, postoperative DVT and postoperative hospital stay.

### Statistical methods

Data analysis was performed *via* SPSS statistical software (version 22.0). The measurement data conforming to the normal distribution is expressed by mean ± standard deviation, and the comparison between groups is by t test. The measurement data that is not normally distributed is expressed by M [P25; P75], and the comparison between groups is by the Mann-Whitney U nonparametric test. The enumeration data were described in the form of the number of cases (percentage), and the chi-square test was used for comparison between groups. *p* < 0.05 was considered statistically significant.

## Results

### The comparison results of preoperative baseline data between the two groups

There was no significant difference in baseline data between the two groups, including age, gender, BMI, disease type, coexisting diabetes, coexisting high blood pressure, APTT, PT, TT, FIB, RBC, HB, HCT, CRP and ESR (*p* > 0.05) in Table 1.

**Table 1:** Comparison of preoperative baseline data between the two groups.

Groups	Observation group (*n* = 23)	Control group (*n* = 21)	t/χ^2^/Z	p
Age, year	55.39 ± 10.95	55.76 ± 9.98	−0.117	0.907
Sex, *n* (%)			0.063	0.802
Male	14 (60.90)	12 (57.10)		
Female	9 (39.10)	9 (42.90)		
BMI, kg/m^2^	24.68 ± 2.09	25.81 ± 3.55	−1.299	0.201
Disease type, *n* (%)			1.001	0.606
Lumbar disc herniation	3 (13.04)	4 (19.05)		
Lumbar spinal stenosis	11 (47.83)	7 (33.33)		
Lumbar spondylolisthesis	9 (39.13)	10 (47.62)		
Coexisting diabetes, *n* (%)	2 (8.70)	3 (14.29)	0.341	0.658
Coexisting high blood pressure, *n* (%)	3 (13.04)	7 (33.33)	2.573	0.155
APTT, s	30.90 ± 2.07	30.85 ± 3.35	0.052	0.959
PT, s	11.29 ± 0.88	11.11 ± 0.78	0.724	0.473
TT, s	15.00 [13.90; 15.60]	15.35 [14.50; 15.81]	−1.458	0.145
FIB, g/L	2.72 [2.52; 3.14]	2.68 [2.36; 3.15]	−0.529	0.597
RBC, 10^12^/L	4.47 [4.10; 4.70]	4.36 [4.16; 4.57]	−0.035	0.972
HB, g/L	143.57 ± 11.33	141.00 ± 10.91	0.763	0.449
HCT, L/L	0.42 [0.38; 0.44]	0.41 [0.38; 0.44]	−0.343	0.731
CRP, mg/L	0 [0; 0.32]	0 [0; 1.03]	−0.597	0.551
ESR, mm/h	12 [4; 29]	11 [5; 22]	−0.494	0.621

### The comparison results of the main observation indicators between the two groups after surgery

RBC, HB and HCT in both groups remained relatively stable after PLIF. The RBC, HB and HCT in the observation group were higher than those in the control group with statistically significant (*p* < 0.05). CRP and ESR in both groups fluctuated to some extent after PLIF. Except the CRP of the observation group on the 7th day after operation was significantly lower than that of the control group (*p* < 0.05), there was no difference in other CRP and ESR between the two groups (*p* > 0.05) in Table 2.

**Table 2:** Comparison of the main observation indicators between the two groups after surgery.

Groups	Observation group (*n* = 23)	Control group (*n* = 21)	t/Z	p
RBC, 10^12^/L				
1st day	3.99 ± 0.43	3.64 ± 0.35	3.039	0.004
4th day	3.87 ± 0.49	3.55 ± 0.44	−2.303	0.021
7th day	4.12 [3.85; 4.29]	3.59 [3.25; 3.72]	−3.278	0.001
Last tested day	4.16 [3.85; 4.26]	3.51 [3.36; 3.98]	−3.325	0.001
HB, g/L				
1st day	128.22 ± 13.03	116.86 ± 10.48	3.199	0.003
4th day	124.65 ± 14.59	114.05 ± 13.50	2.504	0.016
7th day	130 [121; 139]	117 [103; 123]	−3.163	0.002
Last tested day	128.26 ± 13.17	115.33 ± 10.41	3.627	0.001
HCT, L/L				
1st day	0.37 ± 0.04	0.34 ± 0.03	3.045	0.004
4th day	0.37 [0.33; 0.4]	0.34 [0.29; 0.36]	−2.415	0.016
7th day	0.37 [0.35; 0.4]	0.33 [0.29; 0.35]	−3.537	0.000
Last tested day	0.37 ± 0.04	0.33 ± 0.04	3.821	0.000
CRP, mg/L				
1st day	13.21 ± 7.98	17.68 ± 9.31	−1.715	0.094
4th day	4.79 [1.86; 15.29]	6.89 [3.43; 31.86]	−1.668	0.095
7th day	1.35 [0; 7.19]	9.87 [1.88; 24.31]	−2.58	0.010
Last tested day	2.8 [0.55; 7.68]	6.58 [2.08; 14.16]	−1.635	0.102
ESR, mm/h				
1st day	8 [3; 14]	5 [2; 13]	−1.088	0.277
4th day	18 [8; 44]	26 [9; 40]	−0.564	0.573
7th day	21 [7; 40]	32 [15; 44]	−1.07	0.290
Last tested day	31 [18; 43]	39 [17; 48]	−0.682	0.495

### The comparison results of the secondary observation indicators between the two groups after surgery

All patients successfully completed the operation, the incision healed well, and there was no DVT after operation. The intraoperative blood loss and postoperative drainage volume of the observation group were lower than those of the control group with statistically significant (*p* < 0.05). There was no significant difference in APTT, PT, TT, FIB of the first postoperative day, and operation time and postoperative hospital stay between the two groups (*p* > 0.05) in Table 3.

**Table 3:** Comparison of secondary observation indicators between the two groups after surgery.

Groups	Observation group (*n* = 23)	Control group (*n* = 21)	t/Z	p
Operation time, min	150.52 ± 24.79	155.48 ± 36.02	−0.535	0.595
Intraoperative blood loss, mL	300 [200; 300]	300 [300; 500]	−2.391	0.017
Postoperative drainage volume, mL	200 [190; 250]	330 [290; 350]	−4.475	0.000
APTT, s	28.78 ± 2.49	28.60 ± 2.71	0.233	0.817
PT, s	12.37 ± 0.99	12.34 ± 0.79	0.115	0.909
TT, s	14.55 ± 1.21	14.61 ± 1.03	−0.169	0.867
FIB, g/L	2.87 [2.55; 3.23]	2.87 [2.61; 3.23]	−0.223	0.823
Postoperative hospital stay, day	13 [10; 14]	14 [8; 15]	−0.024	0.981

## Discussion

Surgical safety is the premise of PLIF. Some scholars worried about that TXA may lead to systemic fibrinolytic system inhibition, relative insufficiency of plasmin system activity, reduced thrombolysis and increased DVT, and even death of patients (23, 24). Moreover, the elderly face a higher risk of DVT after surgery (25). Therefore, it is necessary to study the safety of ivTXA in PLIF. Therefore, the study observed preoperative and postoperative coagulation markers (including APTT, PT, TT, FIB) and DVT. The results found that ivTXA in PLIF had no effect on coagulation markers and did not produce DVT. This illustrates the feasibility and safety of our study.

Studies found that ivTXA can reduce perioperative blood loss in PILF (26–28). This study similarly found that a unit dose of ivTXA was effective in reducing intraoperative blood loss and postoperative drainage in PILF. It was reported that the application of TXA in PILF can also reduce the operation time (29). But this study did not yield such results, which is similar to the study of Wang et al. (30). There are two possible reasons for the different outcomes. Firstly, in the present study, cases are all single-segment, and the operation is relatively less difficult, so that the operation time will not be significantly affected. Secondly, the study data is a small sample, which may produce bias.

The study showed that RBC, HB and HCT in both groups remained relatively stable after surgery, but those of the observation group were significantly higher than those of the control group at different times after surgery. It shows that the application of ivTXA can play a role in protecting postoperative blood volume. In addition, elderly patients have poor hematopoietic function, and their ability to correct anemia is relatively weak. Therefore, when there is malnutrition after the operation, the patient is difficult to recover. Studies have shown that due to the influence of surgical trauma, inflammatory reaction, and pain stimulation, patients often suffer from lack of energy, poor appetite, and insufficient intake after surgery, which is not conducive to the correction of postoperative anemia. Meanwhile the anemia and hypoproteinemia can also cause and aggravate gastrointestinal mucosal edema, anorexia and other problems which further aggravates anemia and hypoproteinemia, leading to a vicious circle eventually (31).

The disaster that worries doctors the most after PLIF is infection. Once infection occurs, it challenges the effect of treatment. Clinically, CRP and ESR are important indicators which are commonly used to monitor and predict lumbar postoperative infection (32). CRP is an acute, non-specific phase protein with a half-life of 15 h and it is less than 10 mg/L in 99% of healthy individuals (33). The elevated CRP is not related to the amount of bleeding, operation time, drugs, age and gender, but is related to the bacterial infection, the type and degree of tissue damage (34). CRP generally peaks in 2 or 3 days after surgery and returns to normal within 2 weeks after surgery, and the magnitude and duration of elevated CRP is proportional to the severity of the injury, which can be 10 to 60 times higher than the normal value (35).

ESR, a relatively sensitive indicator, lacks specificity for the diagnosis of infection, but has high sensitivity in the occurrence and development of inflammation (36). The elevated ESR is not related to age, disease type, or blood transfusion, but is related to gender, surgical site, and surgical time (35). ESR will increase within 1 week after lumbar spine surgery, peak on the 4th day, and return to the baseline level in 2 or 3 weeks, but it is more than 25 mm/h in most cases (36). However, ESR has a certain false-positive rate, a positive result does not indicate infection, and its positive predictive value is low, which makes it not valuable for early detection of infection, so it is not appropriate to use ESR alone to monitor postoperative infection (35).

Therefore, continuously observation of CRP and ESR after PLIF can accurately determine whether infection occurs, which is conducive to timely detection and treatment of infection. However, there are few studies on postoperative CRP and ESR when TXA is used clinically in PLIF. In addition, different studies hold different viewpoints. Peng Zhao et al. (16) observed the CRP and ESR on the 3rd day after PLIF with ivTXA, and found that they were no different from the group without TXA. Jianru Yuan et al. (17) found that ivTXA could reduce CRP on the 3rd day after PLIF, which may be related to the ability of TXA to relieve postoperative inflammatory response, but the specific mechanism is still unclear. The present study found that CRP and ESR after PLIF with or without ivTXA both fluctuated to some extent. And only the CRP of the observation group was significantly lower than that of the control group only on the 7th day after operation, and there was no difference between the two groups at other time points, which was in line with the general change regulation of CRP and ESR after operation. The possible reason is that the control group continued to have low levels of anemia indicators after surgery, resulting in a weaker ability to fight inflammatory responses, manifested as high CRP on the 7th day after surgery. Therefore, it was possible to speculate that a unit dose of ivTXA in PLIF does not affect the post-operative inflammatory response process.

Enhanced recovery after surgery (ERAS) is a good strategy for patients (37), and studies have shown that the application of TXA is beneficial to ERAS (22). Reducing perioperative blood loss can promote wound healing and early postoperative recovery (38). Theoretically, the postoperative hospital stay should be shortened in patients receiving TXA. However, this study did not yield the similar result, which is similar to the research results of some scholars (39, 40). There are two possible reasons for this. First, the hospital adopts standardized treatment and high-quality nursing measures, which may make no difference in the postoperative hospital stay of patients. Second, the sample size of the study is small, which may cause bias, which cannot be ignored.

## Conclusion

There are some deficiencies in this study, one is the retrospective analysis of medical records, and the other is the small sample size, so the reliability of the conclusions is inevitably affected to some extent. In sum, we believe that the application of unit dose of ivTXA in PLIF has the characteristics of simple operation, safe and effective in reducing blood loss. Our previous questions in introduction are solved, and it can maintain higher levels of postoperative RBC, HB, and HCT, and does not significantly interfere with the levels of CRP and ESR.

## Data Availability

The original contributions presented in the study are included in the article/Supplementary Material, further inquiries can be directed to the corresponding author.
